# Enduring the Value of Open Radical Prostatectomy in the Era of Robotics: A Single-Center Experience

**DOI:** 10.7759/cureus.98999

**Published:** 2025-12-11

**Authors:** Akil Latief, Abdul Rouf Khawaja, Sajjad A Para, Sajad A Malik, Saqib Mehdi, Arif Hamid

**Affiliations:** 1 Urology, Sher-i-Kashmir Institute of Medical Sciences, Srinagar, IND

**Keywords:** continence, iief-5 score, open retropubic radical prostatectomy, sexual function, trifecta outcome

## Abstract

Introduction: Prostate cancer is a significant global health challenge, being the second most common cancer diagnosed in men worldwide. Radical prostatectomy is the standard treatment for localized prostate cancer, be it open, laparoscopic, or robot-assisted.

Aim: While robotic surgery is dominating the era, understanding the outcomes of open retropubic radical prostatectomy for organ-confined prostate cancers in resource-limited countries and centers with no robot facility is crucial.

Methods: A retrospective study was conducted at Sher-i-Kashmir Institute of Medical Sciences between 2013 and 2023, focusing on patients who underwent retropubic radical prostatectomy. The study included a comprehensive patient workup, such as age, comorbidities, serum prostate-specific antigen levels, digital rectal examination, multi-parametric magnetic resonance imaging of the prostate, prostatic biopsy (Gleason's score), bone scan, and optional prostate-specific membrane antigen (PSMA) positron emission tomography (PET) scan. Intraoperative and pathological variables, including T stage, nodal status, extra-prostatic extension, apical margin involvement, bladder neck status, seminal vesical invasion, lymph nodal status, and postoperative outcomes such as biochemical recurrence and the need for hormonal and salvage radiotherapy, were recorded. Follow-up assessments emphasized trifecta outcomes, focusing on cancer control, urinary continence, erectile function, and overall satisfaction.

Results: Out of 84 patients, continence was achieved by 28 (33%) patients at one month, 73 (87%) at three months, 75 (89%) at six months, and 81 (96%) at 12 months, with only three (3.5%) patients remaining incontinent at one year. Among the 32 (27%) patients who underwent nerve-sparing retropubic radical prostatectomy, 20 (62.5%) were potent with phosphodiesterase type 5 (PDE-5) inhibitor assistance at six months, and all regained potency at one year. Biochemical recurrence occurred in three (3.5%) patients, all of whom received hormonal and salvage radiotherapy. Eighty-one (96%) patients remained disease-free at the last follow-up.

Conclusion: Radical prostatectomy remains a widely accepted treatment for organ-confined prostatic carcinoma. As long as the trifecta is satisfactory, it seems meaningless whether performed by minimally invasive or open surgery, particularly when the affordability and nonavailability of robotic surgery are limiting factors. Such a subset of patients, when operated on by open retropubic radical prostatectomy, enjoys good personal satisfaction with similar oncological outcomes as achieved by robotics.

## Introduction

Prostate cancer presents a significant global health challenge, ranking as the second most frequently diagnosed cancer in men worldwide and the fifth most common cancer overall [1]. Approximately 21% of newly diagnosed cancers in men are prostate cancer cases. The risk of prostate cancer is 70% higher in Black men than in White men, although the reasons for this difference are not yet known [2]. Furthermore, prostate cancer ranks as the sixth leading cause of cancer-related deaths in men. By 2020, prostate cancer was projected to experience the largest proportional increase in cancer cases among men worldwide. The widespread adoption of prostate-specific antigen (PSA) screening has led to increased detection rates of clinically localized prostate cancer, particularly among younger and healthier men, thereby enhancing post-treatment longevity [3]. Radical prostatectomy, radiotherapy, cryotherapy, and high-intensity focused ultrasound (HIFU) are all common ways to treat localized prostate cancer with the goal of curing it. During radical prostatectomy, the entire prostate gland is surgically removed.

The standard treatment for localized prostate cancer is radical prostatectomy. Historically, the gold standard approach was open radical prostatectomy utilizing a retropubic approach. Young is credited with doing the first perineal radical prostatectomy in 1903 [4], and Millin is credited with doing the first retropubic radical prostatectomy (RRP) in 1947 [5]. Walsh et al. were the first to introduce the concept of nerve-sparing RRP in 1982 [6]. Over time, less invasive procedures like laparoscopic radical prostatectomy (LRP) and robot-assisted radical prostatectomy (RARP) became more common. These procedures are the next step in surgical practice. Research has shown that RARP and open RRP have similar oncological outcomes and positive margin rates [7]. However, the high cost and limited accessibility of RARP pose challenges, particularly in developing countries, where open surgery remains a viable option. While there is a false belief that RARP offers better surgical control and good quality of life, we decided to study the same parameters in our patients operated on by open radical prostatectomy due to the nonavailability of a robot at our center. The aim of the current study is to evaluate the outcome of open radical prostatectomy in resource-limited countries and centers where the affordability and availability of robots are in question.

## Materials and methods

In this study, we evaluated the safety, perioperative outcomes, functional outcomes, and oncological outcomes, with specific emphasis on trifecta outcomes, and overall satisfaction following RRP. This retrospective study was conducted at the Department of Urology, Sher-i-Kashmir Institute of Medical Sciences, Srinagar, India, with surgeries performed between 2013 and 2023. The study cohort comprised 84 patients. Patient evaluation included the assessment of age, comorbidities, serum PSA levels, digital rectal examination, multi-parametric magnetic resonance imaging of the prostate, prostatic biopsy (Gleason's score), bone scan, and optional prostate-specific membrane antigen (PSMA) positron emission tomography (PET) scan.

Data collection and analysis

All data was stored in an MS Excel spreadsheet (Microsoft Corporation, Redmond, Washington, United States). Statistical analysis was done by using R (R Foundation for Statistical Computing, Vienna, Austria). All categorical variables were observed as numbers and percentages. Continuous data was assessed for normality of distribution, and parametric and non-parametric tests were applied. All results were discussed at the 5% level of significance.

Surgical procedure

**Figure 1:**
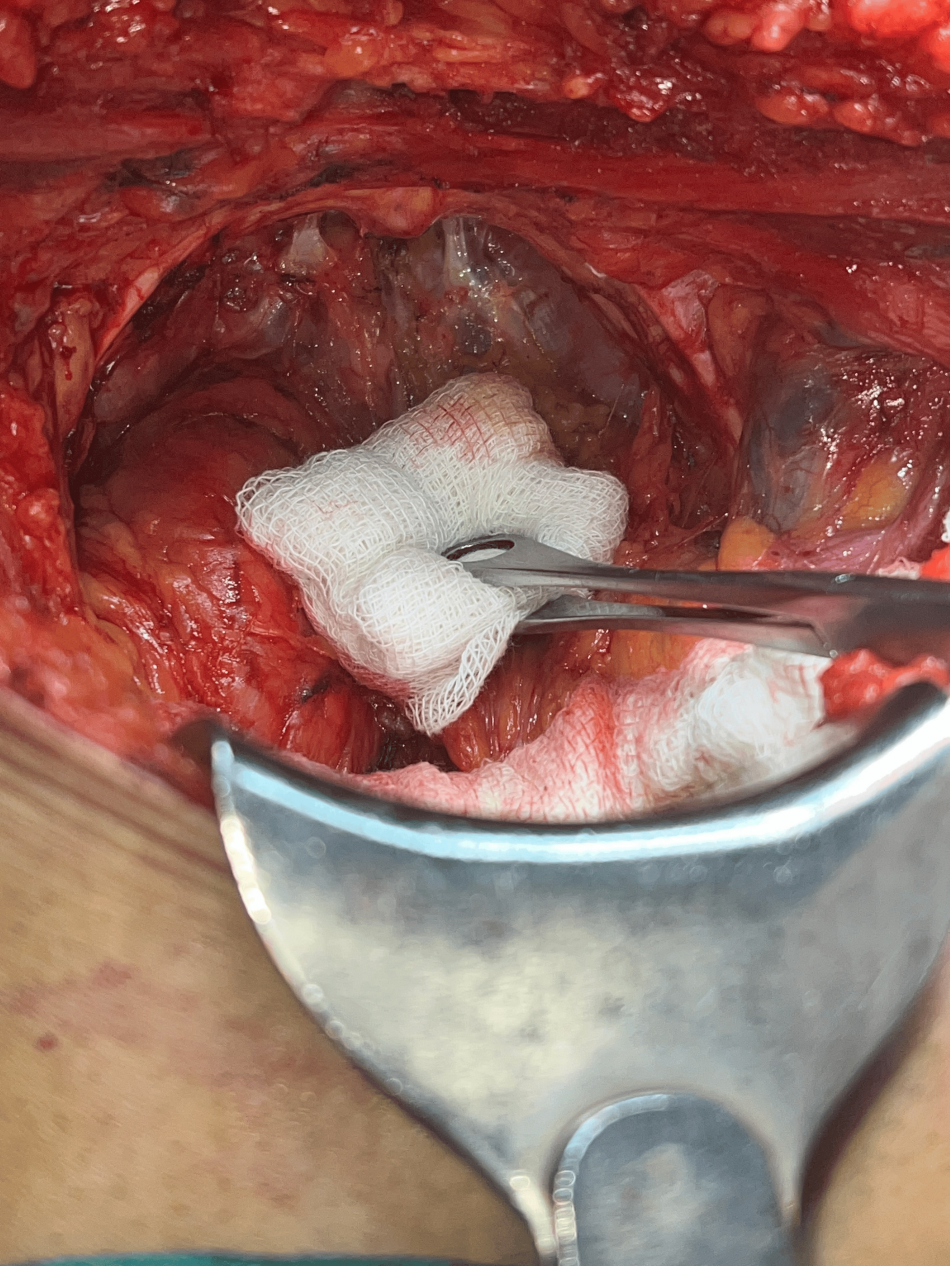
Dorsal venous complex and bilateral puboprostatic ligaments

**Figure 2:**
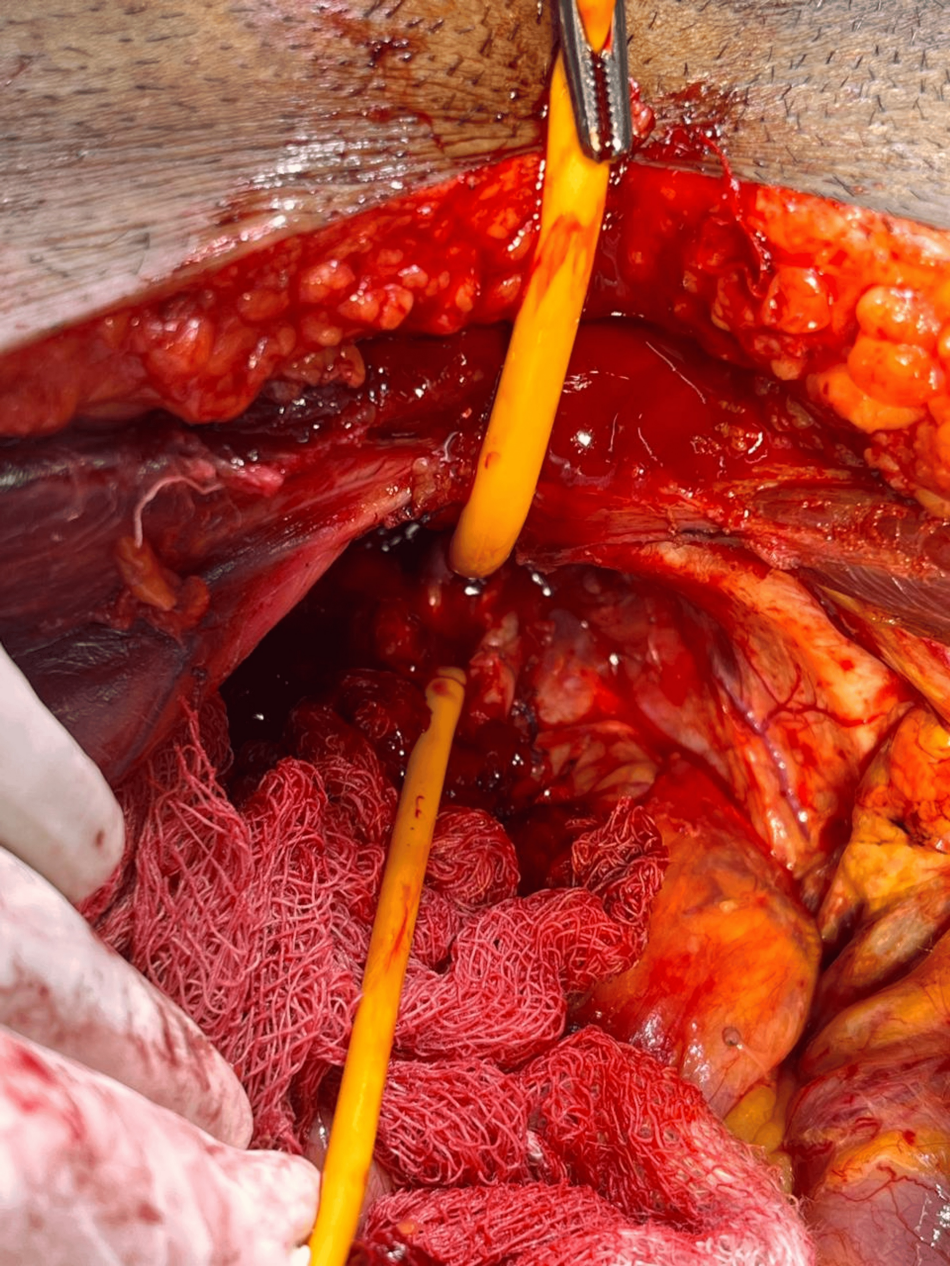
Prostatic apex and urethral transection

**Figure 3:**
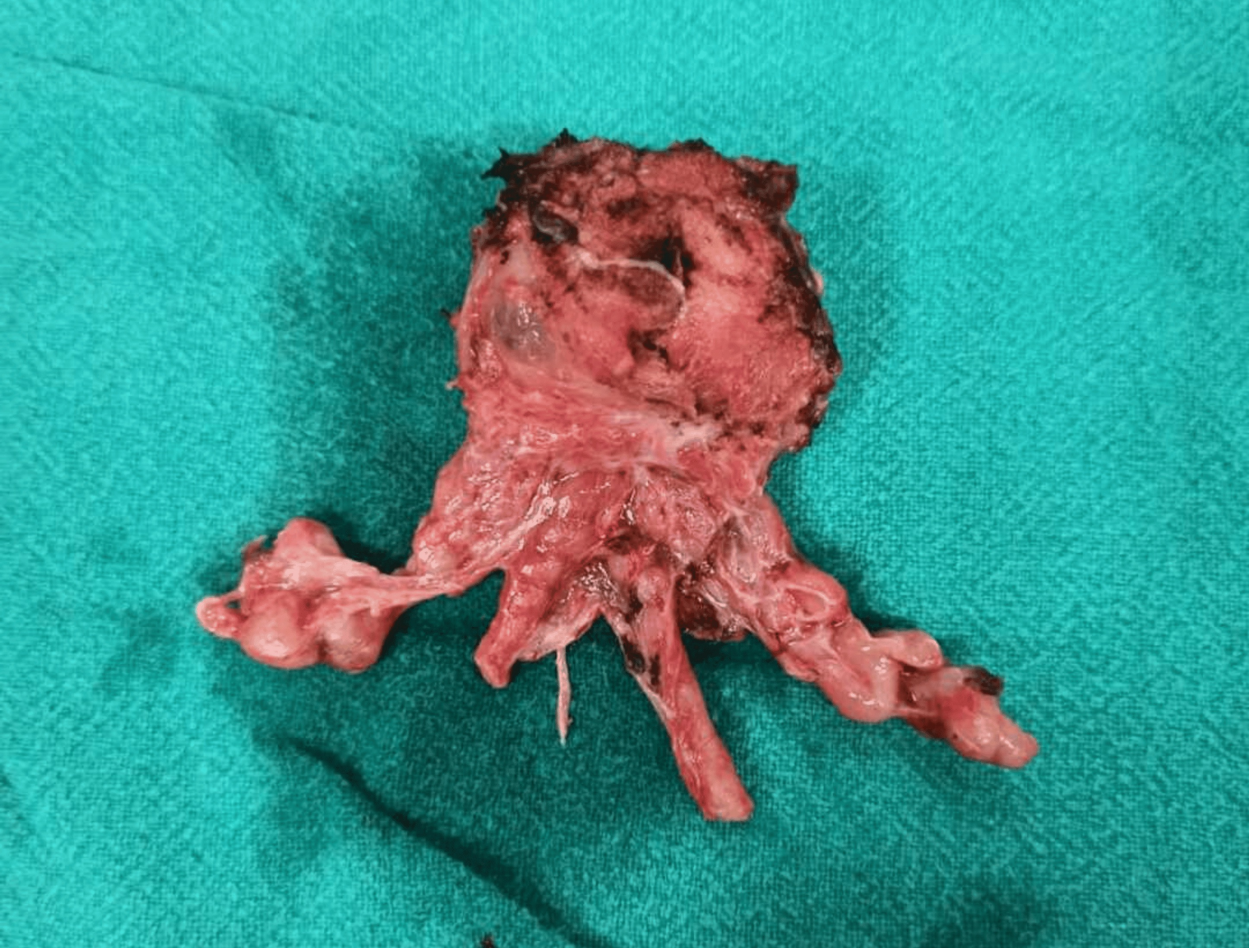
Prostate specimen with bilateral seminal vesicles and vas deferens

Following comprehensive preoperative evaluations and anesthesia clearances, patients were prepared for surgery. The procedures were conducted with patients positioned supine, with a 15-degree tilt at the pelvic level. The majority of surgeries were performed under general anesthesia and a few under regional to mitigate the risks associated with general anesthesia. An infra-umbilical midline incision was made in all cases. Furthermore, standard pelvic lymphadenectomy was done in all cases. Incision was followed by dissection of the pre-vesical space, removal of the retropubic fat, and opening of the endopelvic fascia. The surgical team transected the puboprostatic ligaments and then applied sutures to the dorsal venous complex (DVC) (see Figure 1) before proceeding with the transection. Careful apical dissection (see Figure 2) was undertaken to minimize the risk of margin positivity. The urethra was transected, and dissection proceeded in a retrograde fashion. Preservation of the neurovascular bundles was prioritized, with meticulous release from the prostate without undue traction during dissection (Figure 3). In cases where the neurovascular bundle appeared involved, en bloc resection was performed along with the specimen. Reconstruction of the bladder neck was performed as necessary using size 3-0 absorbable sutures in required cases. Urethro-vesical anastomosis was accomplished using eight size 3-0 monocryl sutures, ensuring a watertight seal over an 18F silicon Foley catheter. Subsequently, a pelvic drain was placed in all cases following the completion of radical prostatectomy.

Due to the unavailability of robotic facilities, open surgery was performed. Intraoperative findings and pathological variables, including T stage, lymph nodal status, apical margin involvement, bladder neck status, presence of extra-prostatic extension, seminal vesicle invasion, postoperative biochemical recurrence, and the need for any hormonal and salvage radiotherapy, were meticulously assessed. During follow-up, particular emphasis was placed on evaluating the trifecta outcomes, focusing on cancer control, urinary continence, erectile function, and overall patient satisfaction.

Postoperative care

**Figure 4:**
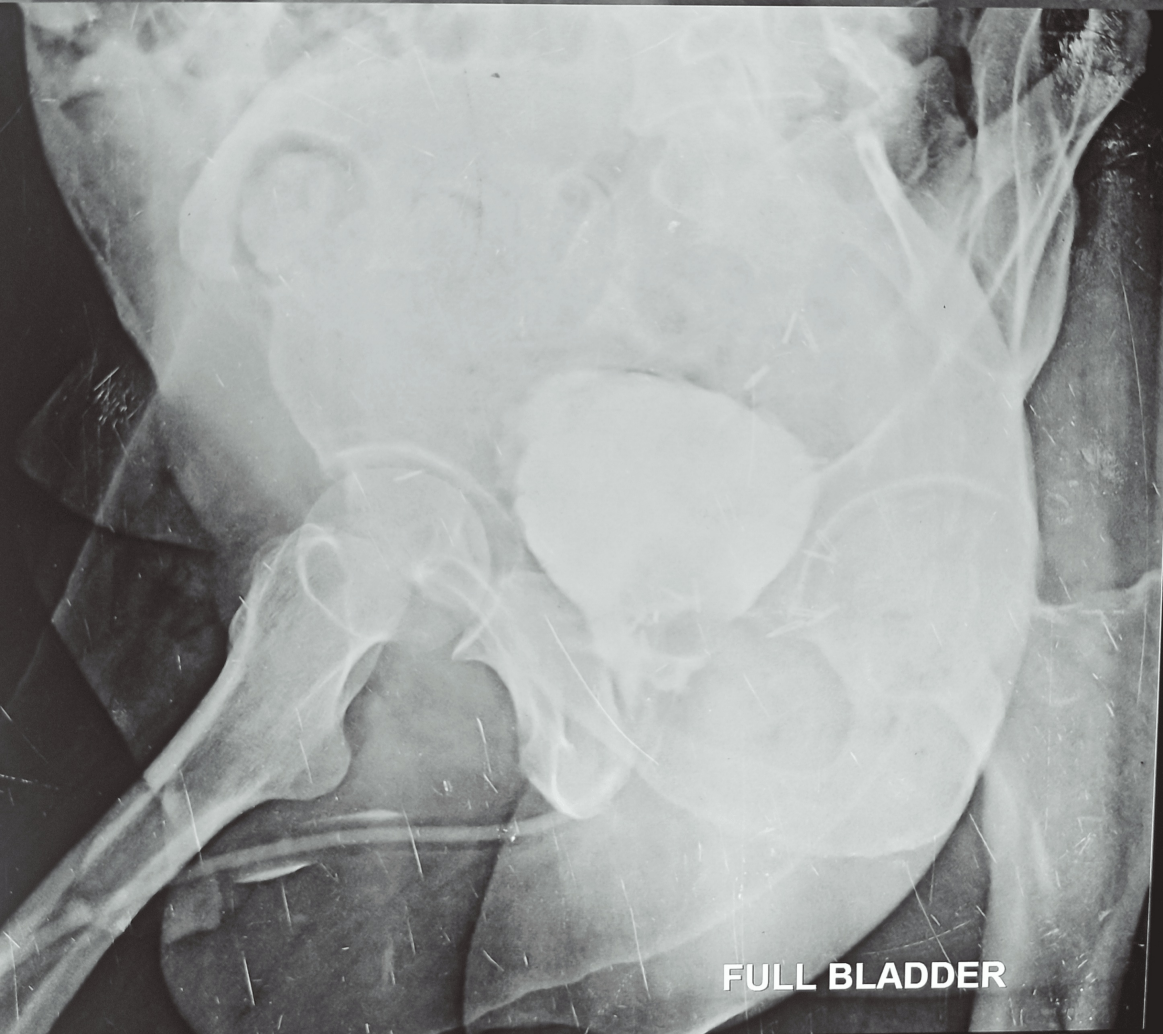
Postoperative peri-catheter study

Following surgery, patients received analgesia using non-opioid agents administered via the epidural or intravenous route, following the hospital's standard postoperative protocol. Patients undergoing RRP were encouraged to ambulate on the first postoperative day. Drain removal occurred once patients passed flatus and had output less than 100 ml in 24 hours. Discharge with an indwelling catheter was facilitated once patients were ambulatory and tolerating a regular diet. Patients returned to the outpatient department initially after one week for suture removal, followed by 21 days post-surgery for catheter removal after doing a peri-catheter study (Figure 4). Continence recovery was assessed on the day of catheter removal and at one, three, six, and 12 months thereafter. Patients were educated about assessing the extent of incontinence during their hospital stay, with reinforcement at catheter removal. On the day of catheter removal, all patients were advised to use adult diapers. Based on urine leakage, they transitioned to protective pads (underwear linings) and recorded the frequency of diaper/pad changes daily. Preoperative International Index of Erectile Function (IIEF-5) scores were obtained through the questionnaire. Patients who were sexually active began a penile rehabilitation program with phosphodiesterase type 5 (PDE-5) inhibitors, and their erectile function was evaluated at six and 12 months using the IIEF-5 questionnaire. Histopathological examination of postoperative specimens was conducted by in-house pathologists, with detailed records of the final Gleason score and margin status.

Postoperative assessment

Postoperative assessment included evaluating positivity, seminal vesicle involvement, and lymph node status. Postoperative PSA levels were monitored at three-month intervals. Biochemical recurrence was defined as two consecutive PSA levels ≥0.2 ng/ml following RRP [8]. Satisfaction was evaluated based on three categories, that is, not satisfied, partially satisfied, and completely satisfied, considering factors such as continence, cancer control, erectile function, and cost.

## Results

The mean age of patients in our study was 62 years (range: 55-74 years). The mean operative time was 190 minutes, and the mean blood loss was 450 ml. Four (5%) patients in our study needed postoperative blood transfusions; two of these patients had low hemoglobin preoperatively. The average hospital stay of the patients was four days. Patients were discharged with a Foley catheter in situ and were asked to report to the outpatient department at three weeks postoperatively for catheter removal. The mean follow-up was 36 months. Post-prostatectomy pathological grading was T2 disease in all cases. A positive surgical margin was seen in 4/84 (Table 1). 

**Table 1:** Demographic, clinical, and pathological profile of patients PSA: prostate-specific antigen; TRUS: trans-rectal ultrasound; RP: radical prostatectomy

Parameter	Value
Age in years (mean, range)	62, 55-74
Age group, n (%)	
50-59 years	30 (36)
60-69 years	51 (60.5)
>70 years	3 (3.5)
Comorbidities, n (%)	
Diabetes mellitus	6 (7)
Hypertension	10 (12)
Presentation, n (%)	
Screening	34 (40.5)
Lower urinary tract symptoms	50 (59.5)
Clinical stage, n (%)	
T1	0 (0)
T2	84 (100)
Serum PSA, n (%)	
4-10 ng/ml	18 (21.5)
11-20 ng/ml	58 (69)
>20 ng/ml	8 (9.5)
Gleason score on TRUS biopsy, n (%)	
3+4	56 (67)
4+3	16 (19)
4+4	12 (14)
Gleason score on RP, n (%)	
3+4	44 (52)
4+3	26 (31)
4+4	14 (17)
Apical margin positivity	4 (4.7)
Extracapsular extension	4 (4.7)
Bladder neck involvement	0 (0)
Seminal vesicle involvement	4 (4.7)

Out of 84 patients, 28 (33%) were continent at the one-month follow-up, 73 (87%) at three months, 75 (89%) patients at six months, and 81 (96%) patients at 12 months. Three (3.5%) patients continued to be incontinent, with one patient among these incontinent patients showing initial improvement with uroflowmetry and cystoscopic findings, followed by worsening at one year. On further evaluation, the same patient had lumbosacral spinal pathology, for which the patient was operated on in the neurosurgery department. Histopathological examination of the lesion revealed ependymoma. Post-surgery, the patient had both bowel and bladder incontinence. Nerve-sparing RRP was done in 32 (27%) patients, with all the patients in the age group of 55-60 years, out of whom 20 (62.5%) patients were potent with PDE-5 inhibitor assistance at six months and all at 12 months. (Potency was defined as the capability to achieve erections sufficient enough for penetration more than 50% of the time.) Three (3.5%) patients had biochemical recurrence on follow-up, and they were subjected to both hormonal and salvage radiotherapy. Biochemical recurrence was defined as two consecutive PSA levels ≥0.2 ng/ml after RRP [8]. Seventy-six (90.5%) patients were disease-free at the last follow-up.

Out of 84 patients, 78 (93%) patients (Table 2) were completely satisfied with respect to continence and cost factor. Three (3.5%) patients with biochemical recurrence were partially satisfied, as they needed postoperative adjuvant radiotherapy and hormonal therapy. All 32 (38%) patients who had opted for a nerve-sparing procedure were completely satisfied with erectile function at the one-year follow-up, while in the rest of the patients, erectile function was not a concern, and they had opted for a non-nerve-sparing procedure. Three (3.5%) patients were not satisfied due to persistent incontinence, with one patient (with a spinal lesion) having persistent bothersome bowel as well as urinary incontinence and not being satisfied at all. The average cost for RRP in our study was around Rs 25000 INR, while for the RARP, the average cost is about Rs 3.5-5 lakh.

**Table 2:** Postoperative continence and satisfaction status

No. of pads used per day	Day 0	1 month		3 months	6 months		12 months
No pad	0	16		45	63		72
1 pad	0	12		26	12		9
2 pads	4	30		6	4		0
3 pads	50	20		3	2		2
4 pads	26	4		2	2		1
5 pads	4	2		2	1		0
Continence status, n (%)	0 (0)	28 (30.3)		73 (76)	75 (89)		81 (96)
Patient satisfaction at one year, n (%)	Completely satisfied		Partially satisfied			Dissatisfied	
	78 (92.8)		3 (3.6)			3 (3.6)	

## Discussion

Radical prostatectomy stands as a cornerstone treatment for localized prostate cancer, with the open radical prostatectomy employing a retropubic approach serving as the gold standard for many years. Young pioneered the perineal approach in 1903 [4], and Millin described the first retropubic approach in 1947 [5]. In 1982, Walsh et al. established the contemporary concept of nerve-sparing RRP [6]. With time, surgical management evolved toward minimally invasive methods such as LRP and RARP. Despite these newer approaches, both RARP and open RRP have been observed to provide comparable cancer control and margin status [7]. However, the limitation of RARP lies in its limited availability and high perioperative costs. The procurement and maintenance of equipment pose additional challenges. The costs associated with RARP are substantial, and during the initial learning curve, the operating time tends to be longer compared to RRP. LRP demands a high level of expertise. While RARP has largely supplanted RRP in developed nations, RRP remains the preferred procedure for prostate carcinoma in developing countries with limited resources and without access to robotic technology. RRP offers advantages such as its feasibility in centers with readily available equipment and its cost-effectiveness. The infra-umbilical incision used in RRP minimizes morbidity, and dissection in the extra-peritoneal plane avoids peritoneal cavity violation. Any urine leak or bleeding is confined to the extraperitoneum, and the absence of gut handling contributes to early bowel activity and reduced hospital stays. In our study, most procedures were performed under combined spinal-epidural anesthesia [9], offering the benefits of both spinal and epidural anesthesia, including rapid onset and profound anesthesia levels, the option for supplementary doses, and effective postoperative pain management, thereby facilitating early postoperative recovery.

Postoperative care considerations

Following RRP with combined spinal-epidural anesthesia, the need for ventilatory support and intensive care unit (ICU) care is obviated. This approach offers added benefits in the current COVID-19 era, as it eliminates the need for sophisticated equipment required for pneumoperitoneum, thereby reducing aerosol formation. Furthermore, avoiding intubation, ventilation, and extubation minimizes aerosol spread and decreases exposure to healthcare personnel [10].

Imaging before surgery and overall satisfaction

All patients underwent thorough preoperative imaging, including multi-parametric magnetic resonance imaging of the prostate, with detailed discussions held with radiologists prior to surgery. Regarding overall satisfaction at one year post-surgery, 78 patients were completely satisfied with respect to continence. However, three patients experiencing biochemical recurrence were partially satisfied, necessitating postoperative adjuvant radiotherapy and hormonal therapy, and three patients who failed to achieve continence at one year were totally not satisfied. Thirty-two patients reported complete satisfaction with erectile function, while those who opted for non-nerve-sparing procedures were not concerned about erectile function.

Instruments and postoperative management

The utilization of specialized instruments aids in procedural ease and enhances oncological outcomes. Instruments such as binocular loops, apical retractors, and bladder neck retractors contribute to improved visualization and help prevent apical and basal margin positivity, respectively. In experienced hands, the rate of apical margin positivity is the same with an open radical prostatectomy, LRP, and RARP [11]. Additionally, a curved needle holder facilitates DVC control, reducing blood loss. Postoperatively, all patients received low-molecular-weight heparin for deep venous thromboprophylaxis, with pulmonary thromboembolism (PTE) prophylaxis initiated 12 hours post-surgery and continued for two weeks [12].

Continence and recovery after surgery

Continence outcomes were favorable at the one-year follow-up due to meticulous dissection, preservation of urethral length, and pre- and postoperative pelvic floor exercises in the form of combined concentration therapy and Kegel's exercise provided by the hospital's Physical Medicine and Rehabilitation (PMR) department. Micturating cystourethrography (MCU) was performed in all patients before Foley catheter removal at two weeks to assess for anastomotic leaks. Delayed catheter removal was implemented if urinary extravasation was detected, aiming to prevent stricture formation.

Oncological management

Patients experiencing biochemical recurrence underwent adjuvant radiotherapy and hormonal therapy, yielding positive responses. Normo-fractionated intensity-modulated radiation therapy (IMRT) was administered to achieve a dose of 70 Gy in 35 fractions. Six months of androgen deprivation therapy (ADT) was added post-radiotherapy. Notably, open radical prostatectomy has demonstrated comparable trifecta outcomes to robotic techniques, with several studies highlighting its cost-effectiveness and efficacy [13-16]. In our study, we observed that all patients who are not dry for one year need evaluation apart from urological intervention, which was seen in our patient as lumbosacral spinal pathology.

We introduce a simple descriptive term for P-R-O-S-T-A-T-E-C-T-O-M-Y: Prostatic radical open surgery needs specialized tools and training in the effective control of the trifecta. Only with limited resources in men's health does it yield comparable results with robotic surgery. We observed from our study that you should choose a surgeon you trust rather than being obsessed with technology like robotics for prostate carcinoma.

Limitations of the study include its retrospective nature and small sample size. Additionally, the lack of a randomized controlled arm and potential selection bias inherent in a single-center series may limit the generalizability of these findings compared to high-volume robotic centers.

## Conclusions

Radical prostatectomy remains a widely accepted treatment for organ-confined prostatic carcinoma. As long as the trifecta is satisfactory, the treatment modality should not limit the timely intervention to the patient, whether performed by minimally invasive or open surgery, particularly when affordability and nonavailability of robotic surgery are factors. Such subsets of patients, by open RRP, enjoy good personal satisfaction with similar oncological outcomes achieved by robotics.
